# Describing the impacts of COVID-19 on the labor market in Japan until June 2020

**DOI:** 10.1007/s42973-021-00081-z

**Published:** 2021-07-19

**Authors:** Taiyo Fukai, Hidehiko Ichimura, Keisuke Kawata

**Affiliations:** 1 Economic and Social Research Institute, Cabinet Office, Government of Japan, Tokyo, Japan; 2 University of Arizona, Tucson, AZ USA; 3 University of Tokyo, Tokyo, Japan

**Keywords:** COVID-19, Job creation, Decomposition, Causal machine learning, J21

## Abstract

The Labor Force Survey, a large-scale government statistics, and the causal forest algorithm are used to estimate the group average treatment effect of the COVID-19 on the employment status for each month from January to June 2020. We find that (1) because of the seasonality in employment status at monthly level, whether we use January 2020 as the base month for comparison, as done in most of the studies or whether we use the same month last year as the base comparison group makes a large difference; (2) whether we include those who are absent from work among the employed or not makes a large difference in the measure of the impact of COVID-19 and its changes; (3) if we use the employment measure which does not include those who are absent from work among the employed, 25–30% among the employed are adversely affected and that 10% of the employed experienced more than 10% decline in employment probability in April, 2020; (4) those who are the most affected by the COVID-19 are those who are unemployed or work part-time in the hotel and restaurant industry and service occupations; (5) in addition, younger and female respondents are more affected than are older and male respondents; and (6) we observe no clear differences in the impacts of COVID-19 with respect to living location, education status, and firm size among the most affected.

## Introduction

COVID-19 has affected the Japanese labor market by directly affecting the behaviors of employers and employees and also by inducing various government policies to restrict social and economic activities in an effort to contain the spread of the virus.

This paper describes how the Japanese labor market was affected by COVID-19 through June 2020. We provide basic facts based on the Labor Force Survey (LFS) using employment status as the outcome measure. The LFS is a monthly household survey conducted by the Ministry of Internal Affairs and Communications (MIC) and has a similar design to the Current Population Survey (CPS) in the United States. The LFS includes approximately 40 thousand households and contains information on individuals’ demographics as well as on the work and education status of those 15 years or older. The large sample size allows us to use data-driven subsample analysis to identify the heterogeneity of the impacts of COVID-19.

Our main interest is the difference of the employment probability in a month and that of the same month in the previous year, conditional on respondents’ background characteristics and working status in the previous month. To avoid misspecification problems, causal machine learning techniques are used, which are useful for estimating not only the average difference but also heterogeneity in terms of background characteristics. First, the difference in the conditional probabilities is estimated by a causal machine learning technique (Wager and Athey 2018; Athey et al. 2019). The result is used to estimate the percentiles of the conditional probabilities. This is used in turn to define the most affected group. We then characterize the most affected group by comparing the difference in the means of various covariates for those among the most affected group and those not among the most affected group. See (Athey et al. 2019; Chernozhukov et al. 2018b).

Our findings are as follows: (1) because of the seasonality in employment status at monthly level, whether we use January as the base month for comparison, as done in most of the studies or whether we use the same month last year as the base comparison group makes a large difference;^1^ (2) whether we include those who are absent from work among the employed or not makes a large difference in the measure of the impact of COVID-19 and its changes; (3) if we use the employment measure which does not include those who are absent from work among the employed, 25–30% among the employed are adversely affected and that 10% of the employed experienced more than 10% decline in employment probability in April, 2020; (4) those who are the most affected by the COVID-19 are those who are unemployed or work part-time in the hotel and restaurant industry and service occupations; (5) in addition, younger and female respondents are more affected than are older and male respondents; and (6) we observe no clear differences in the impacts of COVID-19 with respect to living location, education status, and firm size among the most affected.

The remainder of the paper is organized as follows. Section 2 reviews what we know so far about the impacts of COVID-19 on labor markets in Japan and other countries. Section 3 explains the data and shows the overall impacts of COVID-19 through June 2020 by the difference between the observed and predicted employment dynamics. Section 4 introduces our empirical framework to discover the heterogeneous effects of COVID-19, and Sect. 5 reports the estimation results. Finally, Sect. 6 concludes the paper.

## Background and related literature

This section provides a brief overview of the spread of COVID-19 infection in Japan in the first half of 2020 and the actions taken by the government. We then review what we know about the impacts of the spread of COVID-19 infection on the labor market from previous studies in Japan and abroad.

### COVID-19 situation in Japan in the first half of 2020

Since the identification of the first case of COVID-19 in early 2020, the infection has been gradually spreading across Japan. By June 2020, in Japan, more than 18,000 people had been infected, and more than 950 people had died.^2^

The Japanese government took various measures to contain the spread of infection. First, measures to reduce human-to-human contact were taken; all elementary, junior high, and high schools in Japan were asked to temporarily close their schools for 1 month starting in March 2020.^3^ Furthermore, in April 2020, as the infection was spreading rapidly, a “state of emergency” was declared. Under this “emergency declaration,” the prefectural governors of the targeted areas could, by law, request residents to refrain from going out, except when necessary to maintain their livelihoods, and to cooperate as necessary to prevent infection. It should be pointed out that these were requests only and not regulations with penalties such as those put into effect during the so-called “lockdown.” Moreover, compensation for absence from work was given to workers who were forced to take leave, and subsidies were extended to those in need. In addition, a policy was adopted to provide 100,000 yen per person to support households.^4^ Subsidies were provided not only to workers but also to business establishments when they requested leave.^5^ These combined policies were expected to make effective use of the “emergency declaration” and reduce the spread of the disease.^6^

**Fig. 1:**
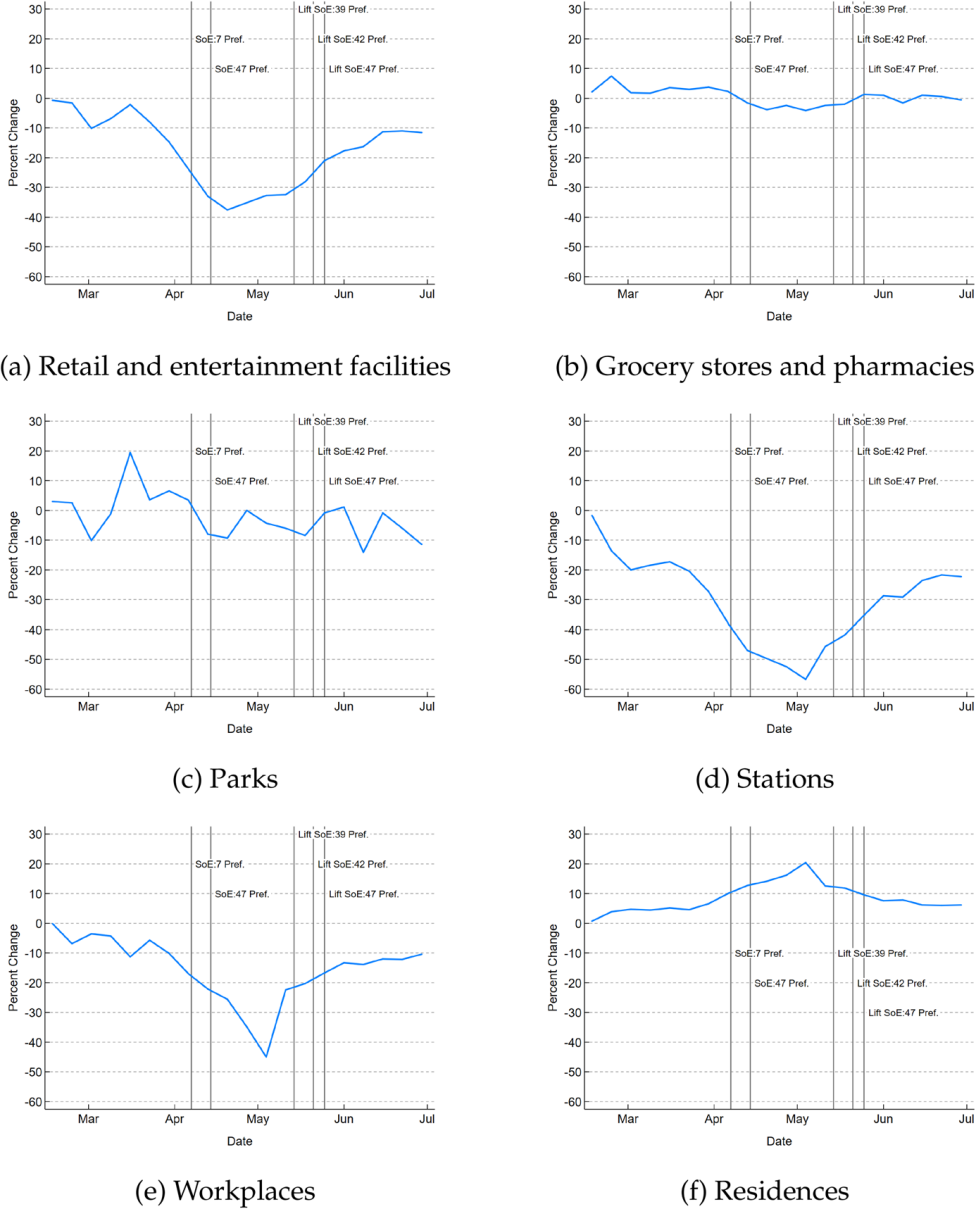
Mobility of people during COVID-19 *Source*: Google LLC “Google COVID-19 Community Mobility Reports”, https://www.google.com/covid19/mobility/ Accessed: 20200918. *Note*: Each figure shows the percentage changes in the number of visitors to (or time spent in) the corresponding category of location from the end of February to the end of June 2020 compared to the day-of-week baseline. The day-of-week baseline is the median value for each day of the week for the 5-week period from January 3 to February 6, 2020. We aggregated daily data to weekly averages. Retail and entertainment facilities include restaurants, cafes, shopping centers, theme parks, museums, libraries, movie theaters, etc. Grocery stores and pharmacies include grocery stores, food wholesalers, fruit and vegetable markets. Parks are national parks, public beaches, marinas, dog parks, squares, gardens, etc. Stations are subway, bus and train stations. SoE is an abbreviation for "State of Emergency". On April 7, 2020, a state of emergency was first declared for Tokyo, Kanagawa, Saitama, Chiba, Osaka, Hyogo and Fukuoka. It was extended to the entire country on April 16. The state of emergency was lifted on May 14, except for eight prefectures: Hokkaido, Tokyo, Kanagawa, Chiba, Saitama, Osaka, Hyogo and Kyoto. On May 21, it was lifted in Osaka, Hyogo, and Kyoto. The state of emergency was lifted in all prefectures on May 25

We can see from the mobility data how these sets of policies affected people’s activities. Mobility measures from “Google COVID-19 Community Mobility Reports” ([17]), Fig. 1 shows the weekly change in the number of visitors to (or time spent in) each category of location from the end of February to the end of June 2020 compared to the day-of-week baseline. The day-of-week baseline is the median value for each day of the week for the 5-week period from January 3 to February 6, 2020, and shows the percentage change in activity for each week compared to activity during this period. The figure indicates that shopping at grocery stores and pharmacies, which are necessary for daily life, decreased slightly during this time but not too much, while trips to shopping centers and entertainment facilities decreased significantly. In addition, the use of train stations and visits to workplaces decreased significantly, indicating that people were spending more time at home. These changes were particularly pronounced in April and May, when the state of emergency was declared, indicating that there were substantial changes in people’s movements and activities during this period.

### Related literature

How has the spread of COVID-19 and the resulting changes to people’s lives affected the labor market? Previous domestic and international studies have reported descriptive results focusing not only on the overall impacts of the novel coronavirus outbreak on the levels of employment, hours, and wages but also on the heterogeneous impacts of the outbreak according to individual and job characteristics. The examined individual characteristics are gender, age, and education, and the examined job characteristics are occupation, industry, whether self-employed or not, the percentage of tasks that can be done from home, and work arrangement (temporary/permanent, salaried/not salaried, and fixed hours/varying hours). Furthermore, whether or not the regional coronavirus prevalence differences are important is also examined.

Adams-Prassl et al. (2020), using a real-time survey with 20,910 respondents, find that while 18% and 15% of respondents reported losing their jobs due to the coronavirus outbreak in the US and the UK, respectively, in April 2020, only 5% reported so in Germany. For the US, the figure is consistent with the 19% reported by Cowan (2020) for the same period using the Current Population Survey (CPS), which added 6 percentage points to the unemployment rate. Cowan (2020) also reports a 3-percentage-point decrease in the labor force participation rate, a 3-percentage-point increase in the rate of absence from work, and a more than 2-percentage-point increase in the likelihood of working part-time when the worker usually works full-time.

In their analysis of heterogeneous impacts, the base group includes male employees, younger than 30 years, who cannot work from home, are without a university degree, and who are working as nonsalaried, temporary workers, with varying hours. This base group experienced job loss probabilities of 43%, 25%, and 13% in the US, the UK, and Germany, respectively. The base employee group is the most affected group, with the exception of a few groups. First, females in the US and UK experienced higher job loss probabilities by 3% and 2%, respectively. Second, workers in their 30s in the UK experienced 3% higher job loss probability compared to the base group. In all three countries, permanent workers have lower job loss probabilities by 6%, 17%, and 5% in the US, the UK, and Germany, respectively. Additionally, in all three countries, those who can work from home have lower job loss probabilities by 26%, 19%, and 4% in the US, the UK, and Germany, respectively. Self-employed individuals in the US and UK have lower job loss probabilities by 10% and 5%, respectively. Salaried workers in the US and Germany have lower job loss probabilities by 6% and 2%, respectively. In all three countries, there are few age differences (except for workers in their 30s in the UK, as discussed above), and there is no difference in terms of whether or not employment involves fixed hours when all other factors described above are controlled.

In the US, workers with a university degree were better off than those without a university degree and experienced 8% lower earning loss probability, and workers 40 years or older experienced 7–10% less earning loss probability, analogously to those workers older than 60 years, and in the UK, workers 50 years or older experienced 6% lower earning loss probability. Only those aged 60 years or older experienced lower earning loss probability in Germany. Workers in the US and UK, whose jobs allow them to work from home entirely, experienced 13% and 8% lower earning loss probabilities, respectively. There was no such effect in Germany. Although self-employed individuals in the US had similar earning loss probability to that of employees, in the UK and Germany, self-employed individuals experienced 10% and 7% higher earning loss probabilities, respectively.

As in these three countries, the impacts of COVID-19 on the labor market in Japan are expected to vary by worker. As Dingel and Neiman (2020) also points out, the effects of COVID-19 can differ between jobs that allow for flexible changes in the work environment and those that do not. Kawaguchi and Motegi (2020) notes that in Japan, teleworking option, which allows individuals to work from home, varies widely across occupations, with higher-skilled and higher-income workers being more likely to take advantage of such schemes. In particular, Kikuchi et al. (2021) points out that nonregular workers and women are more likely to be in jobs that require much interpersonal contact and are difficult to work remotely, which points to the potential higher impacts of the spread of the disease on these workers.

## Data and descriptive evidence

This study uses individual-level data from the Labor Force Survey (LFS) in Japan from January 2013 to June 2020. The LFS is a monthly survey on forty thousand households from stratified regions throughout Japan, and all household members aged 15 years and older are required to respond. The survey collects detailed information on labor, such as employment status, position in the workforce, firm size, industry, and occupation, and demographics, such as household composition, age, and gender, including on household members under the age of 15 years.

The LFS is a rotating sample. Every household that enters the LFS is surveyed for 2 months, left alone for ten months, and then surveyed again for two more months. Every month, one-fourth (10,000) of new households enter the rotation and one-fourth leave the rotation. At the end of the second 2-month survey, each household answers an additional special questionnaire, which asks, among other questions, about annual income and information about the previous job if the individual is not working. In this paper, we exploit the 2-month panel structure to examine the transition of employment status.

In this paper, we focus on whether or not respondents are working, i.e., their employment status. Employment in the LFS includes those who are working and those who are absent from work. The latter in the LFS is assumed to include individuals on leave with salary compensation, such as long-term care leave and parental leave. However, the responses under the setting of COVID-19 may include absence from work without salary compensation due to the temporary closure of the workplace. For this reason, we examine employment with and without considering absence from work in employment. To express which outcome measure is used succinctly, we refer to the employment measure inclusive of workers who are absent from work as *the loose employment* measure and that excluding workers who are absent from work as *the strict employment* measure.

We first characterize the overall impact of COVID-19 on the Japanese labor market. To control for seasonality and to control for the differences in the improving trend in employment rates among males and females for different age groups prior to the COVID-19 outbreak, we use the following regression equation and examine the outcome as the deviation from the predicted value constructed from this equation for each group, thus coefficients are allowed to differ across the groups:

1 $$\begin{aligned} y_{imt} = \beta _0 + x_{imt}'\beta + \mu _m + f(t) + u_{imt}, \end{aligned}$$

where *i* is the individual, *m* is the survey month, and *t* indicates the time when the year and month are converted to continuous variables; explanatory variable, *x*, includes dummy variables for gender and 5-year age group dummy variables; $\mu _m$ is month fixed effects, which account for seasonality in employment status; and *f*(*t*) controls for the linear time trend in the employment rate. In our analysis, we estimate the regression Eq. (1) using data from 2013 to 2018. Using the estimated coefficients, we calculate the predicted employment rate from 2013 to June 2020. Then, the predicted employment rate for each individual is aggregated by month to provide an overview of the changes to the employment rate due to COVID-19. Note that we did not use 2019 data in our predictions to check whether our predictions are appropriate by comparing them with the actual employment rate in 2019, when COVID-19 had not yet been identified.

**Fig. 2:**
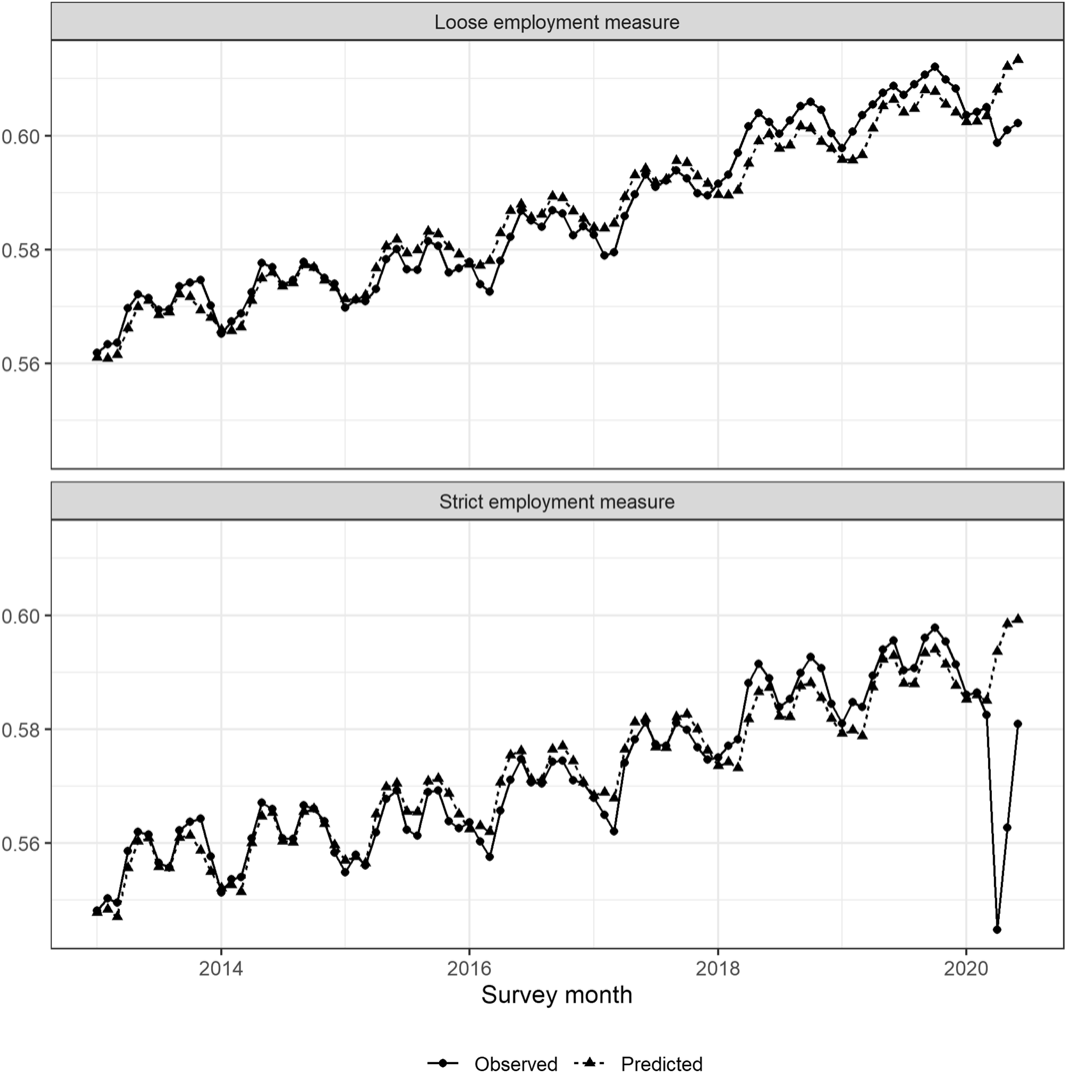
Observed and predicted employment rates *Sources*: The Labor Force Survey (MIC) *Notes*: This figure shows the predicted employment rates with the employment rate observed from the data. The upper panel uses the strict employment measure, while the lower panel uses the loose employment measure. The predicted values are estimated from Eq. 1 by the OLS estimator using the data from January 2013 to December 2018. We refer to the employment measure inclusive of workers who are absent from work as *the loose employment* measure and that excluding workers who are absent from work as *the strict employment* measure

Figure 2 compares the predicted employment rate outcome with the employment rate observed from the data. The upper panel of Fig. 2 uses the strict employment measure, while the lower panel uses the loose employment measure. The solid line is the observed employment rate, and the dotted line is the predicted value estimated from Eq. 1 by the OLS estimator using data from January 2013 to December 2018. The predicted values from Eq. (1) and the observed employment rates closely match, so our predictions seem appropriate. In particular, data for 2019, when COVID-19 infection had not yet spread, are not used in the prediction, but the estimated results predict the observed values well. This result reinforces our claim that Eq. (1) predicts the employment rate well in the absence of the spread of COVID-19.

In Fig. 2, we see that the employment rates of both measures are clearly below the predicted values from April to June 2020, during the spread of COVID-19 infection in Japan. The results are more pronounced when the strict employment measure is used, with the employment rate dropping by four percentage points in April. After that, there was some recovery in May and June but not a full recovery. When the loose employment measure is used, the employment rate drops by only slightly more than one percentage point in April 2020. The recovery trend is not pronounced with this indicator, and it remains low in May and June. These results indicate that the number of lost workdays and individuals who are unemployed or out of the labor force were higher April to June of 2020, when the infection spread, compared to previous years, and that the number of lost workdays was much higher in April. While the impact on the lost workdays in May and June is smaller compared to that in April, it still exists, and the impact on individuals who are unemployed or out of the labor force remains higher in May and June 2020 than in other years.

We formalize the graphical analysis through the following regression analysis, which is a slight modification of regression Eq. (1).

2 $$\begin{aligned} y_{imt} = \beta _0 + x_{imt}'\beta + \sum _{\begin{array}{c} l\in \{Jan19, \\ \ldots , Jun20\} \end{array}}\delta _l D(t = l) + \mu _m + f(t) + u_{imt}, \end{aligned}$$

**Fig. 3:**
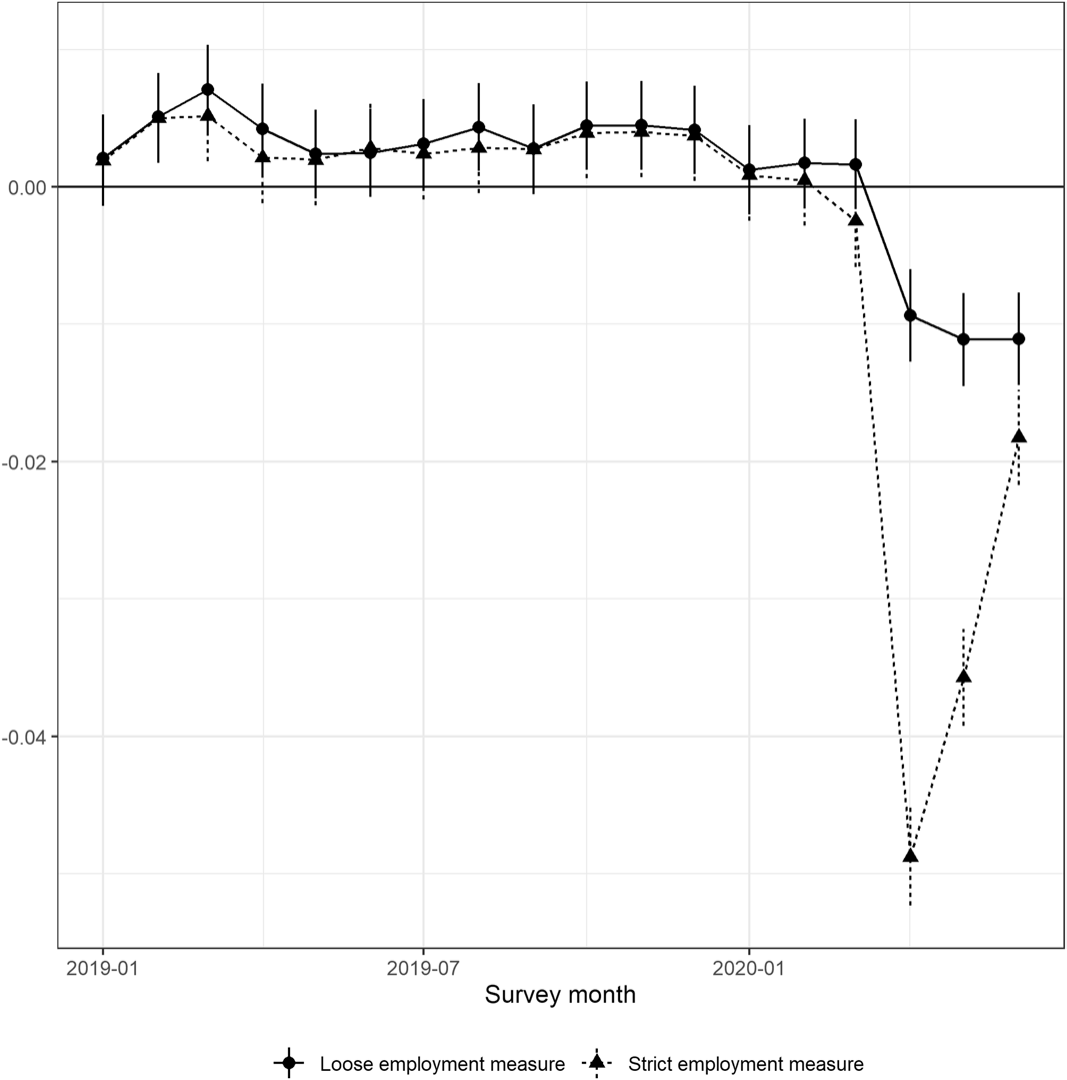
Gap between observed and predicted employment rates *Sources*: The Labor Force Survey (MIC) *Notes*: This figure shows the deviation from the employment rate predicted by the attributes and trends for each month from January to June 2020, $\delta _l$ in Eq. 2. Each dot reports the coefficient in each month, and the bars are the 95% confidence intervals. We refer to the employment measure inclusive of workers who are absent from work as *the loose employment* measure and that excluding workers who are absent from work as *the strict employment* measure

We make our estimations using all data from 2013 to June 2020; $\delta _l$ captures the deviation from the employment rate predicted by the attributes and trends for each month from January 2019 to June 2020.^7^^,^^8^ Figure 3 reports the overall changes in the loose and strict employment measures between January 2019 and June 2020.

**Table 1:** Specification check Source: The Labor Force Survey (MIC)

Indep. var	Loose employment measure				Strict employment measure			
Mean in 2019	0.6040				0.5881			
	(1)	(2)	(3)	(4)	(5)	(6)	(7)	(8)
2020.01	0.0012	0.0012	0.0014	0.0014	0.0009	0.0009	0.0009	0.0009
	(0.0017)	(0.0017)	(0.0017)	(0.0016)	(0.0017)	(0.0017)	(0.0017)	(0.0017)
2020.02	0.0017	0.0017	0.0019	0.0019	0.0005	0.0005	0.0005	0.0005
	(0.0016)	(0.0016)	(0.0016)	(0.0016)	(0.0017)	(0.0017)	(0.0017)	(0.0017)
2020.03	0.0016	0.0016	0.0018	0.0018	– 0.0025	− 0.0025	− 0.0024	− 0.0024
	(0.0017)	(0.0017)	(0.0017)	(0.0017)	(0.0017)	(0.0017)	(0.0017)	(0.0017)
2020.04	− 0.0094$$^{***}$$	− 0.0093$$^{***}$$	− 0.0092$$^{***}$$	– 0.0092$$^{***}$$	− 0.0488$$^{***}$$	-0.0488$$^{***}$$	− 0.0487$$^{***}$$	− 0.0487$$^{***}$$
	(0.0017)	(0.0017)	(0.0017)	(0.0017)	(0.0018)	(0.0018)	(0.0018)	(0.0018)
2020.05	− 0.0111$$^{***}$$	− 0.0111$$^{***}$$	− 0.0109$$^{***}$$	− 0.0109$$^{***}$$	− 0.0357$$^{***}$$	− 0.0357$$^{***}$$	− 0.0357$$^{***}$$	− 0.0357$$^{***}$$
	(0.0017)	(0.0017)	(0.0017)	(0.0017)	(0.0018)	(0.0018)	(0.0018)	(0.0018)
2020.06	− 0.0111$$^{***}$$	− 0.0110$$^{***}$$	− 0.0109$$^{***}$$	− 0.0109$$^{***}$$	− 0.0182$$^{***}$$	− 0.0182$$^{***}$$	− 0.0182$$^{***}$$	− 0.0182$$^{***}$$
	(0.0017)	(0.0017)	(0.0017)	(0.0017)	(0.0018)	(0.0018)	(0.0018)	(0.0018)
Specific trend								
Sex	N	Y	N	N	N	Y	N	N
Age	N	N	Y	N	N	N	Y	N
Sex and Age	N	N	N	Y	N	N	N	Y
$N$	7214280	7214280	7214280	7214280	7214280	7214280	7214280	7214280

This table shows the coefficients (deviations from the predicted values) for each month in 2020, as estimated in Eq. 2. We include dummy variables for gender and 5-year age group dummy variables as independent variables. Robust standard errors are in parentheses. We refer to the employment measure inclusive of workers who are absent from work as *the loose employment* measure and that excluding workers who are absent from work as *the strict employment* measure. For the mean in 2019 for each employment rate, the average from January to June is used. $$^{*}$$ $$\textit{p}<0.1$$, $$^{**}$$ $$\textit{p}<0.05$$, $$^{***}$$ $$\textit{p}<0.01$$

**Table 2:** Sensitivity check *Source*: The Labor Force Survey (MIC)

Indep. var	Loose employment measure			Strict employment measure		
Mean in 2019	0.6040			0.5881		
Sample period	2013–2020	2014–2020	2015–2020	2013–2020	2014–2020	2015–2020
	(1)	(2)	(3)	(4)	(5)	(6)
2020.01	0.0012	− 0.0011	−0.0055$$^{***}$$	0.0009	− 0.0014	− 0.0054$$^{***}$$
	(0.0017)	(0.0017)	(0.0018)	(0.0017)	(0.0017)	(0.0018)
2020.02	0.0017	− 0.0003	−0.0040$$^{**}$$	0.0005	− 0.0015	− 0.0049$$^{***}$$
	(0.0016)	(0.0017)	(0.0018)	(0.0017)	(0.0017)	(0.0018)
2020.03	0.0016	− 0.0004	− 0.0040$$^{**}$$	−0.0025	− 0.0043$$^{**}$$	− 0.0073$$^{***}$$
	(0.0017)	(0.0017)	(0.0018)	(0.0017)	(0.0018)	(0.0018)
2020.04	− 0.0094$$^{***}$$	− 0.0112$$^{***}$$	− 0.0149$$^{***}$$	− 0.0488$$^{***}$$	− 0.0505$$^{***}$$	− 0.0539$$^{***}$$
	(0.0017)	(0.0018)	(0.0018)	(0.0018)	(0.0019)	(0.0019)
2020.05	− 0.0111$$^{***}$$	− 0.0132$$^{***}$$	− 0.0167$$^{***}$$	− 0.0357$$^{***}$$	− 0.0377$$^{***}$$	− 0.0408$$^{***}$$
	(0.0017)	(0.0018)	(0.0018)	(0.0018)	(0.0018)	(0.0019)
2020.06	− 0.0111$$^{***}$$	– 0.0135$$^{***}$$	− 0.0175$$^{***}$$	− 0.0182$$^{***}$$	− 0.0205$$^{***}$$	– 0.0240$$^{***}$$
	(0.0017)	(0.0018)	(0.0018)	(0.0018)	(0.0018)	(0.0019)
$N$	7214280	6221083	5250929	7214280	6221083	5250929

This table shows the coefficients (deviations from the predicted values) for each month in 2020, as estimated in Eq. 2. We include dummy variables for gender and 5-year age group dummy variables as independent variables. Robust standard errors are in parentheses. We refer to the employment measure inclusive of workers who are absent from work as *the loose employment* measure and that excluding workers who are absent from work as *the strict employment* measure. For the mean in 2019 for each employment rate, the average from January to June is used. $$^{*}$$ $$\textit{p}<0.1$$, $$^{**}$$ $$\textit{p}<0.05$$, $$^{***}$$ $$\textit{p}<0.01$$

By inspecting the 95% confidence intervals drawn in the figure, we see that all coefficients for April to June 2020 are statistically significant. The strict employment rate drops by approximately five percentage points in April 2020 and then recovers slowly, and by June 2020, the employment rate is lower by approximately two percentage points. These results indicate the following three points. First, when the state of emergency was declared in April, workers were affected by taking leaves of absence, becoming unemployed or being out of the labor force. Second, those who were affected by taking leaves of absence constitute a large fraction of those affected. Third, those who took leaves of absence in April recovered in May and June, but not fully. The impact on those who were unemployed and out of the labor force may not have improved in May and June. It should also be pointed out that the coefficients before 2020 are mostly stable throughout 2019, indicating that our prediction is appropriate. In addition, the coefficients of the first month in 2020 are close to zero, suggesting that the COVID-19 shock hit the labor market as it was deteriorating.

Using the same framework, we will examine the heterogeneity of the impacts of COVID-19 by running regression Eq. (2) by gender and age.

**Fig. 4:**
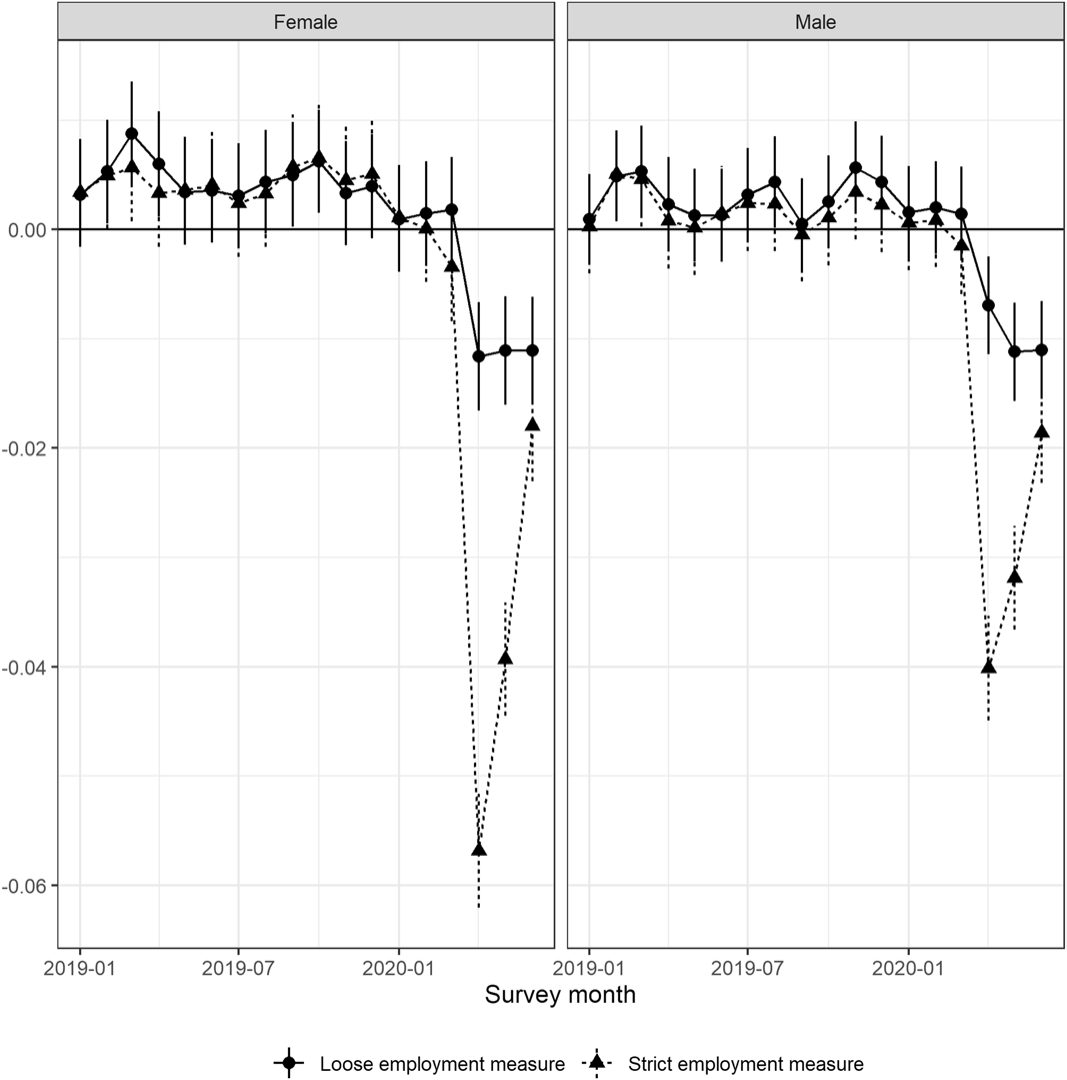
Gap between observed and predicted employment rates: Gender *Sources*: The Labor Force Survey (MIC) *Notes*: This figure shows the deviation from the employment rate predicted by the attributes and trends for each month from January to June 2020 by gender. Each dot reports the coefficient in each month, and the bars are the 95% confidence intervals. We refer to the employment measure inclusive of workers who are absent from work as *the loose employment* measure and that excluding workers who are absent from work as *the strict employment* measure

Figure 4 shows the deviation from previous years in terms of the employment rate, broken down by gender. The left panel shows the results for females, and the right panel shows the results for males. Overall, there is no significant difference between males and females, except in April 2020, when the impact on females was approximately 1.8 percentage points larger than that on males, with the strict measure of employment. The rest of the results are the same as the previous results.

We further examine employment by age in addition to gender.

**Fig. 5:**
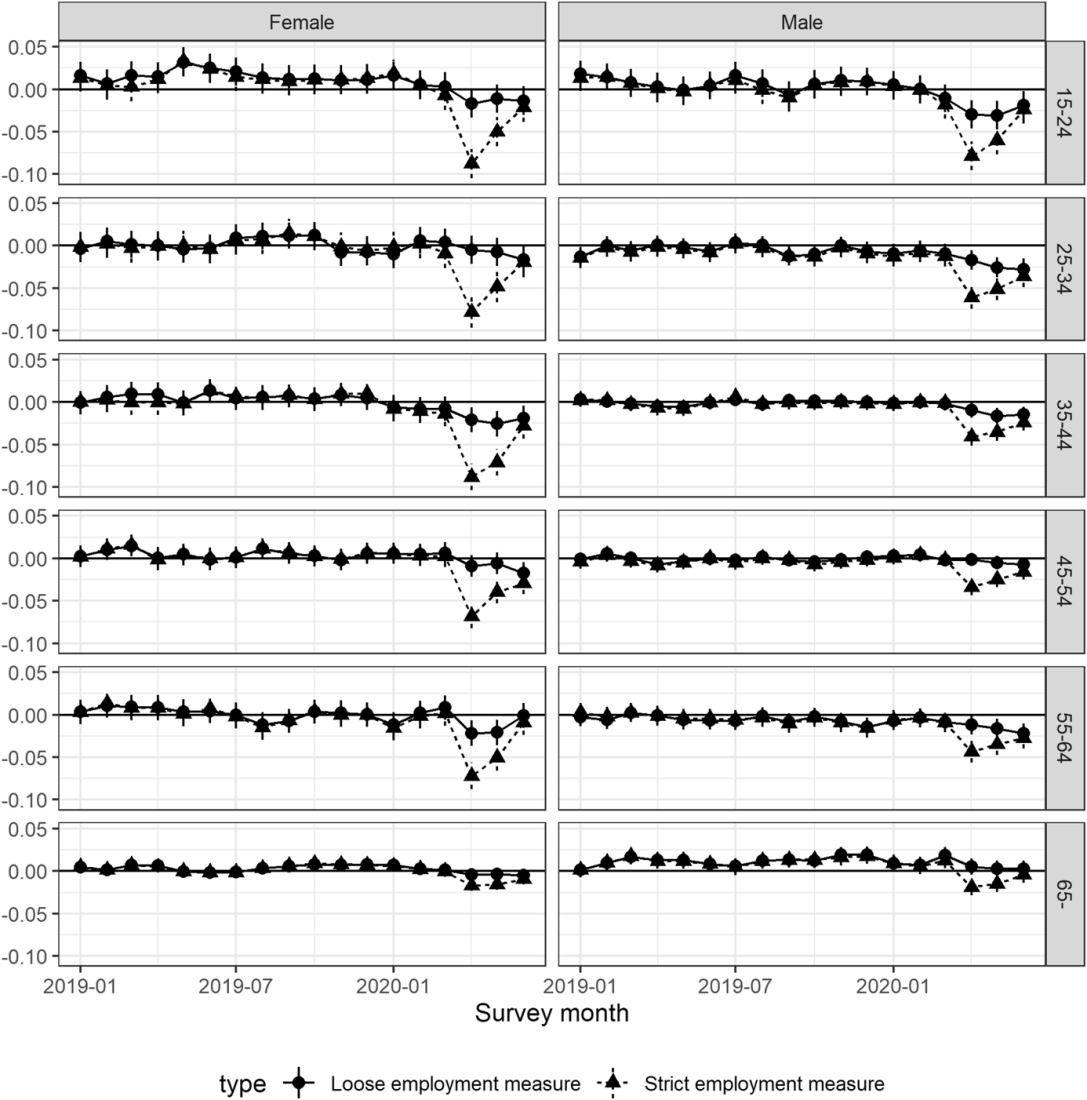
Gap between observed and predicted employment rates: Gender and age *Sources*: The Labor Force Survey (MIC) *Notes*: This figure shows the deviation from the employment rate predicted by the attributes and trends for each month from January to June 2020 by gender and age group. Each dot reports the coefficient in each month, and the bars are the 95% confidence intervals. We refer to the employment measure inclusive of workers who are absent from work as *the loose employment* measure and that excluding workers who are absent from work as *the strict employment* measure

Figure 5 shows the estimated impact on the employment rate by age and gender. The impacts of COVID-19 on the strict employment measure are particularly large among females given their age, except for those aged 64 years or older. Moreover, the impacts on the younger generation (under 35 years) and women under 65 years are particularly large, which implies that workers in these groups were more absent from work in April 2020 compared to those in other groups.

When we use the loose measure of employment, the outlook is somewhat different for males than for females. While this measure also indicates that those who are 65 years or older were not affected very much, except for a few groups (those below 25 years and females 55–64 years old), this measure indicates that the impact was getting worse, not better, from April to June 2020.

## Estimation method

We have shown that the impacts of COVID-19 vary by age and gender. In the same way, we would like to examine the heterogeneous impacts of COVID-19 on employment by considering many other observable factors, such as industry, job type, and job location.

Formally, let $Y_{1it}$ and $Y_{0it}$ be the employment status indicator with and without the influence of COVID-19, respectively, for individual *i* in month *t*. Both $Y_{1it}$ and $Y_{0it}$ equal one if the respondent is employed and zero otherwise. The interest is $\tau (x)$, which is defined as follows:

3 $$\begin{aligned} \tau (x)=E[Y_{1it} - Y_{0it}|X_{i} = x] \end{aligned}$$

where $X_{i}$ is a vector of respondent characteristics, including age, gender, education, location, family structure, employment type, position, industry, and occupation.

We observe $Y_{1it}$ directly in 2020 for each month but do not observe the counterfactual outcome in 2020 without the impacts of COVID-19, $Y_{0it}$. One approach is to use observations based on 2013–2019 to create a counterfactual outcome for 2020. However, the approach requires us to assume that the trend up to the end of 2019 continues into the first 6 months of 2020. In this study, rather than extrapolating the outcome, we simply compare the outcomes in 2020 with those in the same months in 2019. That is, we estimate $E(Y_{0it}|X_{i}=x)$ by the predicted value given the various characteristics of workers using the month *t* observations in 2019, which, in other words, assumes no trend. That is, we assume $\tau (X)=E[Y|2020,X]-E[Y|2019,X]$, where *Y* is an observed employment status indicator.

### Group average difference

The conditional average difference $\tau (X)$ captures ceteris paribus year-over-year differences given baseline covariate vector *X*. The Labor Force Survey includes many covariates, allowing us to discover many heterogeneous effects, potentially.

A traditional method for estimating $\tau (X)$ uses the linear regression model with interaction terms or subsample analysis. Using the linear regression model is a reasonable approach, especially with a limited sample size. However, this simple method may suffer from some problems. First, the linear model may be misspecified, and as a result, the estimator of $\tau (X)$ may be inconsistent. Second, choosing subgroups ex post, may lead to over-fitting. The popular approach focuses on limited covariates, for instance, focusing on only a few regressors such as gender and age. While this approach is reasonable with a limited sample size, important heterogeneity may be missed.

These problems are serious problems in most empirical studies because no theories ensure the parametric form to consider. In the context of labor market studies, there is no theory indicating the parametric form of labor supply and demand.

This paper uses a semiparametric estimation with machine learning, which is relevant in the context of a large sample size and a large number of covariates. The machine learning tool has recently been applied in empirical analysis to avoid the risks of misspecification and over-fitting (Athey and Imbens 2019). This paper uses the causal forest algorithm, which extends the random forest algorithm (Breiman 2001) to estimate the conditional average difference and reports the sorted group average treatment difference (Chernozhukov et al. 2018a).^9^

The disadvantage of the flexible functional form approach is the difficulty in reporting $\tau (X)$. The sorted group average treatment effect can be viewed as a way to summarize $\tau (X)$.

Formally, the sorted group average treatment difference is defined with $G_1,...,G_{20}$, which is a partition of the support of $X_{i}$. The group average difference ${\bar{\tau }}_l$ is defined as ${\bar{\tau }}_l = E[\tau (x)|x \in G_l]$ for $l=1,\ldots ,20$. We define $\tau _0 = -\infty$ and $x \in G_l$ if and only if $\tau (x)\in (\tau _{l-1},\tau _{l}]$, where we take $\tau _l$ for $l=1,\ldots , 20$ to be the $5\times l$th-quantile value of $\tau (X_{i})$.

1. Estimate $\tau (x)$ (denoted as ${{\hat{\tau }}}(x)$).
2. Make subgroups $G_1,...,G_{20}$ based on ${{\hat{\tau }}}(x)$.
3. Estimate the group average difference ${\bar{\tau }}_l = E[\tau (X)|X \in G_l]$ for $l=1,\ldots ,20$.

We next characterize the group of individuals affected the most by COVID-19, i.e., those in $G_1$. Formally, our interest is as follows:

4 $$\begin{aligned} E[X_{i}|X_i \in G_1]-E[X_{i}|X_i \notin G_1], \end{aligned}$$

where $G_1$ is an individual in the lower fifth percentile in terms of the impacts of COVID-19 on employment status.

The approach allows us to investigate high dimensional covariate vector *X* without the over-fitting problem and helps us discover characteristics of workers who are affected the most by the COVID-19.

### Estimation implementation

To estimate $\tau (x)$, causal forests (Wager and Athey 2018) and (Athey et al. 2019) are employed. Let $I_i(2020)$ be a year indicator, which equals one if respondent *i* was surveyed in 2020 and zero if she/he was surveyed in 2019. Their framework estimates the following model: dropping the month *t* subscript,

5 $$\begin{aligned} Y_i=\tau (X_i)\times I_i(2020)+f(X_i)+u_i, \end{aligned}$$

where $f(x)=E(Y_{i}|X_i=x, I_i(2020)=0)$, and $E[u_i|X_i,I_i(2020)]=0$ is assumed. $\tau (x)$ is the target function, which is estimated as follows:

6 $$\begin{aligned} {{\hat{\tau }}}(x) = \frac{\sum _i \alpha _i(x)[Y_i - {\hat{f}}_Y(X_i)][I_i(2020)-\hat{f}_I(X_i)]}{\sum \alpha _i(x)[I_i(2020) - \hat{f}_I(X_i)]^2}, \end{aligned}$$

where $\alpha _i$ is a data-adaptive kernel weight estimated by the random forest algorithm and $\hat{f}_Y(X_i)$ and $\hat{f}_I(X_i)$ are estimators of $E(Y_i|X_i)$ and $E(I_i(2020)|X_i)$, respectively, also estimated by the random forest algorithm. Estimated groups $\{{\hat{G}}_1,...,{\hat{G}}_{20}\}$ are constructed based on ${{\hat{\tau }}}(X)$.

The average impact in group *l* is estimated by a variant of the double robust estimator (Athey et al. 2019). Formally, for $l=1,\ldots ,20$,

$$\begin{aligned} {\hat{\tau _l}} = \frac{1}{n_l}\sum _{X_i\in {\hat{G}}_l,}{\hat{\Gamma _i}}, \end{aligned}$$

where $n_l$ is the sample size of group ${\hat{G}}_l$,

$$\begin{aligned} {\hat{\Gamma _i}} = {\hat{\tau }}^{(-i)}(X_i) + \frac{I_i-{\hat{f}}^{(-i)}_I(X_i)}{f^{(-i)}_I(X_i)[1-f^{(-i)}_I(X_i)]} [Y_i-f^{(-i)}_Y(X_i)-{\hat{\tau }}^{(-i)}(X_i)(I_i-{\hat{f}}^{(-i)}_I(X_i)], \end{aligned}$$

where the estimators with superscript $(-i)$ are the random forest estimators constructed without using $(Y_i,I_i)$. Finally, the classification analysis is simply implemented by examining the sample analog of

7 $$\begin{aligned} E[X_{i}|X_i\in G_1]-E[X_{i}|X_i \notin G_1]. \end{aligned}$$

## Estimation results

### Estimated group average difference

We first report the estimated group average impacts of COVID-19 for ${\hat{\tau }}_l$ for $l=1,\ldots , 20$ and focus on the most affected group, $G_1$.

**Fig. 6:**
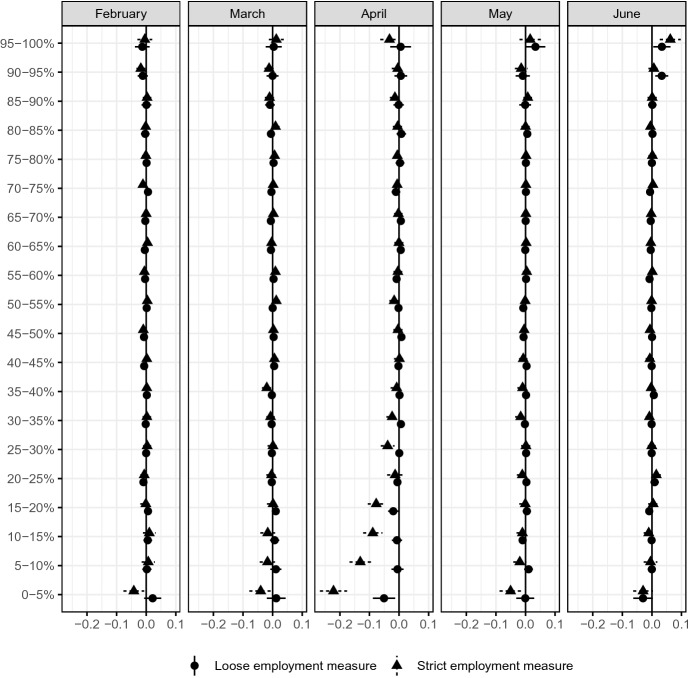
Group average difference compared to the same month in the previous year *Source*: The Labor Force Survey (MIC). *Notes*: This figure shows the group average difference with the strict and loose employment rate in each month. The difference are estimated from Eq. 6 by the AIPW estimator using the data from February 2019 to June 2020. Each dot is a point estimate, and the bars are the 95% confidence intervals. For instance, “5–10%” reports the group average difference of respondents whose $\tau (x)$ is between the 5–10% quantile

**Table 3:** GATE for the loose employment measure *Source*: The Labor Force Survey (MIC)

group	February	March	April	May	June
95–100%	− 0.005 (0.013)	0.012 (0.013)	− 0.032 (0.016)**	0.016 (0.018)	0.062 (0.018)***
90–95%	− 0.018 (0.008)**	− 0.012 (0.008)	− 0.004 (0.01)	−0.014 (0.011)	0.007 (0.009)
85–90%	0.003 (0.007)	− 0.010 (0.008)	− 0.014 (0.009)	0.008 (0.007)	0.001 (0.008)
80–85%	− 0.002 (0.005)	0.010 (0.007)	− 0.005 (0.008)	0.000 (0.007)	− 0.005 (0.006)
75–80%	− 0.001 (0.006)	0.007 (0.006)	− 0.006 (0.005)	0.002 (0.006)	0.002 (0.007)
70–75%	− 0.011 (0.005)**	0.002 (0.006)	− 0.006 (0.005)	0.001 (0.004)	0.003 (0.006)
65–70%	0.000 (0.005)	0.003 (0.005)	− 0.002 (0.007)	0.001 (0.004)	− 0.002 (0.004)
60–65%	0.004 (0.003)	− 0.004 (0.004)	0.000 (0.008)	0.002 (0.004)	− 0.004 (0.004)
55–60%	− 0.006 (0.003)*	0.009 (0.006)	− 0.004 (0.008)	0.004 (0.007)	0.001 (0.005)
50–55%	0.004 (0.004)	0.012 (0.006)*	− 0.016 (0.008)**	− 0.001 (0.006)	− 0.001 (0.006)
45–50%	− 0.010 (0.004)***	0.003 (0.005)	− 0.004 (0.008)	− 0.005 (0.006)	− 0.007 (0.005)
40–45%	0.002 (0.003)	0.006 (0.006)	0.001 (0.009)	− 0.008 (0.007)	– 0.007 (0.007)
35–40%	0.002 (0.006)	− 0.020 (0.008)**	− 0.008 (0.01)	− 0.010 (0.009)	−0.003 (0.006)
30–35%	0.002 (0.007)	− 0.007 (0.007)	− 0.023 (0.01)**	− 0.016 (0.009)*	− 0.008 (0.006)
25–30%	0.003 (0.007)	0.001 (0.009)	− 0.038 (0.012)***	0.001 (0.008)	− 0.001 (0.007)
20–25%	− 0.007 (0.007)	− 0.004 (0.009)	− 0.013 (0.014)	− 0.011 (0.009)	0.015 (0.008)*
15–20%	− 0.001 (0.01)	0.002 (0.01)	− 0.077 (0.014)***	− 0.001 (0.01)	0.004 (0.009)
10–15%	0.011 (0.011)	− 0.017 (0.013)	− 0.089 (0.017)***	− 0.010 (0.01)	− 0.011 (0.009)
5–10%	0.007 (0.011)	− 0.017 (0.013)	− 0.131 (0.018)***	− 0.019 (0.011)*	− 0.005 (0.012)
0–5%	− 0.042 (0.018)**	− 0.040 (0.02)**	− 0.222 (0.023)***	− 0.050 (0.019)***	− 0.030 (0.017)*

This table shows the group average difference with the loose employment measure in each month. The difference are estimated from Eq. 6 by the AIPW estimator using the data from February 2019 to June 2020. We refer to the employment measure inclusive of workers who are absent from work as *the loose employment* measure. $$^{*}$$ $$\textit{p}<0.1$$, $$^{**}$$ $$\textit{p}<0.05$$, $$^{***}$$ $$\textit{p}<0.01$$

**Table 4:** GATE for the strict employment measure *Source*: The Labor Force Survey (MIC)

group	February	March	April	May	June
95–100%	−0.013 (0.013)	0.004 (0.014)	0.005 (0.018)	0.034 (0.017)**	0.034 (0.015)**
90–95%	−0.012 (0.009)	0.000 (0.010)	0.006 (0.011)	−0.009 (0.012)	0.033 (0.011)***
85–90%	0.001 (0.008)	−0.009 (0.008)	−0.001 (0.009)	−0.001 (0.010)	0.001 (0.007)
80–85%	−0.003 (0.007)	−0.006 (0.007)	0.008 (0.008)	0.006 (0.007)	0.002 (0.007)
75–80%	0.001 (0.005)	0.003 (0.007)	0.003 (0.007)	0.001 (0.006)	0.000 (0.006)
70–75%	0.006 (0.004)*	−0.004 (0.004)	−0.011 (0.007)	0.001 (0.006)	−0.006 (0.005)
65–70%	−0.003 (0.005)	−0.006 (0.004)	0.006 (0.005)	0.001 (0.006)	−0.004 (0.004)
60–65%	−0.005 (0.003)	−0.006 (0.004)	0.006 (0.006)	−0.001 (0.005)	−0.004 (0.004)
55–60%	−0.004 (0.002)	0.003 (0.004)	−0.008 (0.006)	0.002 (0.005)	−0.008 (0.005)*
50–55%	0.002 (0.003)	0.000 (0.003)	−0.002 (0.005)	−0.007 (0.003)**	−0.002 (0.003)
45–50%	−0.007 (0.005)	0.003 (0.004)	0.008 (0.006)	−0.006 (0.005)	0.000 (0.004)
40–45%	−0.007 (0.004)*	0.005 (0.005)	−0.002 (0.006)	0.004 (0.004)	−0.001 (0.004)
35–40%	0.002 (0.004)	−0.003 (0.005)	0.002 (0.007)	0.002 (0.005)	0.006 (0.005)
30–35%	−0.002 (0.003)	−0.003 (0.006)	0.006 (0.005)	−0.002 (0.006)	−0.001 (0.005)
25–30%	0.000 (0.005)	−0.003 (0.005)	0.001 (0.006)	0.002 (0.005)	−0.001 (0.005)
20–25%	−0.009 (0.005)*	−0.003 (0.006)	−0.005 (0.006)	0.003 (0.004)	0.009 (0.007)
15–20%	0.006 (0.005)	0.011 (0.006)*	−0.020 (0.009)**	0.005 (0.007)	−0.009 (0.005)**
10–15%	0.005 (0.007)	0.007 (0.008)	−0.007 (0.009)	−0.010 (0.006)	−0.001 (0.006)
5–10%	0.002 (0.008)	0.011 (0.010)	−0.005 (0.010)	0.010 (0.008)	0.000 (0.007)
0–5%	0.022 (0.015)	0.012 (0.016)	−0.051 (0.019)***	−0.001 (0.015)	−0.030 (0.017)*

This table shows the group average difference with the strict employment measure in each month. The difference are estimated from equation 6 by the AIPW estimator using the data from February 2019 to June 2020. We refer to the employment measure excluding workers who are absent from work as *the strict employment* measure. $$^{*}$$ $$\textit{p}<0.1$$, $$^{**}$$ $$\textit{p}<0.05$$, $$^{***}$$ $$\textit{p}<0.01$$

Tables 3 and 4 report the GATE estimates for both strict and loose employment measures, and Fig. 6 presents a graphical summary. We find that a 7.7- to 22.2-percentage-point decline in employment probability is experienced by approximately 20% of potential workers when the strict employment measure is used. In the loose employment measure, the impact is a 5.1-percentage-point decline in April for approximately 5% of potential workers. The strict measure indicates that the impact persists in May (1.9- to 5-percentage-point decline for 10% of potential workers) and June (3-percentage-point decline for 5% of potential workers).

The impacts of COVID-19, as measured by employment status, are concentrated in some respondents. In April, the impact is felt by 35% of potential workers using the strict employment measure, but the extent of the impact is reduced to 10% of potential workers in May and further reduced to 5% of potential workers in June. Using the loose employment measure, the extent of the impact is concentrated among 5% of potential workers, but the impact seems to persist into June.

### Characterization of the most affected group

We next characterize the most affected group, $G_1$. The following figures report the estimated difference in each set of variables in $X_{i}$ in Eq. 7. The positive estimate implies that the average value of the variable in $X_{i}$ is larger in $G_1$ than the average values in the other groups.

**Fig. 7:**
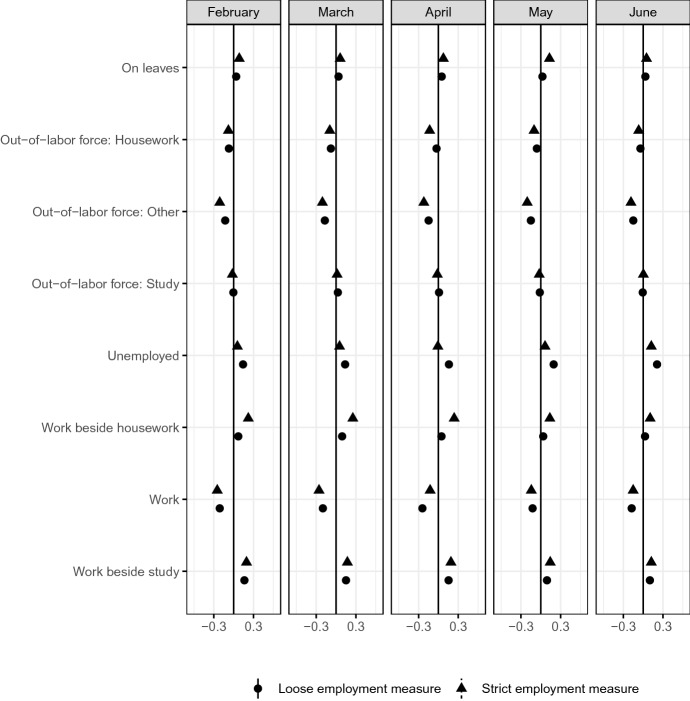
Work status *Source*: The Labor Force Survey (MIC). *Notes*: This figure shows the results of classification analysis in terms of work status in the last month. The difference of the bottom 5% and other groups are estimated from Eq. 7 by the difference-in-means estimator using the data from February 2019 to June 2020. Each dot is a point estimate, and the bars are the 95% confidence intervals

Figure 7 reports the distribution of employment status in the previous month. In both definitions of employment, the mean values of job status in the previous month are significantly different between the most affected group and other groups. In either definition of employment, the most affected group contains a larger share of those who work and study than do other groups. The share of respondents who primarily work is smaller among the most affected group compared to other groups. Using the strict employment measure, the share of respondents who work and do housework, is larger in the most affected group than in the other groups. Using the loose employment measure, the share of unemployed workers among the most affected group, is larger than in the most affect group than in the other groups.

**Fig. 8:**
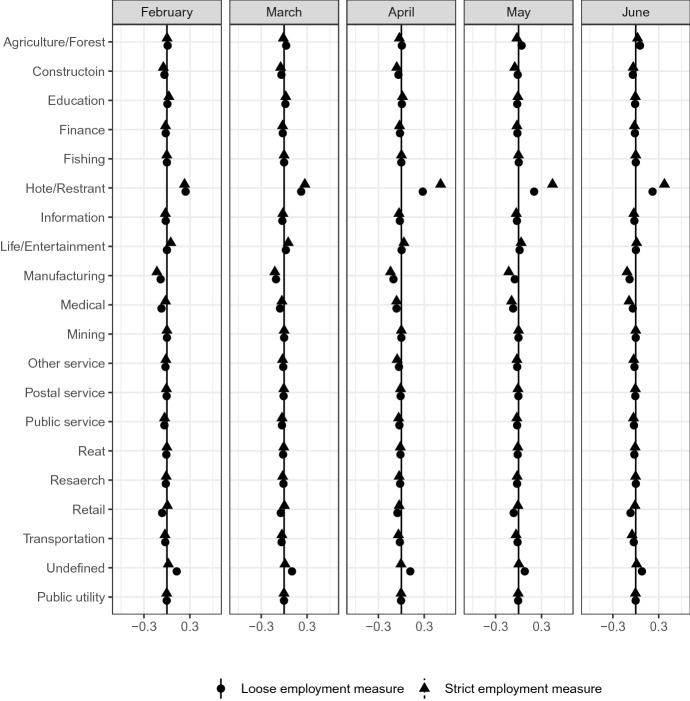
Industry *Source*: The Labor Force Survey (MIC). *Notes*: This figure shows the results of classification analysis in terms of respondent’s industry in the last month. The difference of the bottom 5% and other groups are estimated from Eq. 7 by the difference-in-means estimator using the data from February 2019 to June 2020. Each dot is a point estimate, and the bars are the 95% confidence intervals

**Fig. 9:**
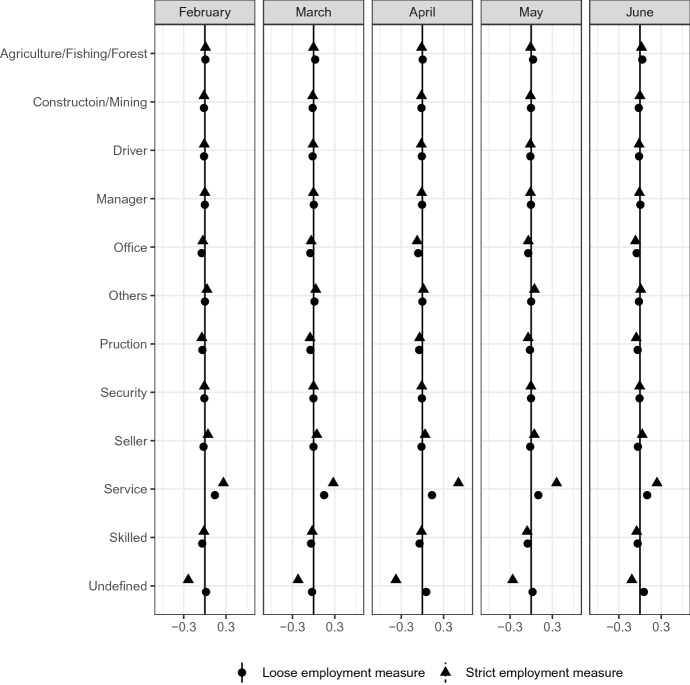
Occupation *Source*: The Labor Force Survey (MIC). *Notes*: This figure shows the results of classification analysis in terms of respondent’s occupation in the last month. The difference of the bottom 5% and other groups are estimated from Eq. 7 by the difference-in-means estimator using the data from February 2019 to June 2020. Each dot is a point estimator, and the bars are the 95% confidence intervals

Figures 8 and 9 report the industry and occupation that respondents worked in over the last month, respectively. (Couch et al. 2020) and (Forsythe et al. 2020) report the heterogeneity of labor market impact across industries and occupations in the United States (US). The following figures report similar results in Japan.

Figure 8 shows that the impacts of COVID-19 are concentrated in the hotel and restaurant industry. The result holds regardless of whether we use the strict or loose measure of employment. The result is noticeable in February and persists into June. In either definition of employment, individuals in the manufacturing industry are relatively less likely to be among the most affected group.

Figure 9 shows that the occupation distribution is also different between the most affected group and other groups. In either definition of employment, respondents in service occupations tend to belong to the most affected group, and the magnitude of the difference is large, starting in February and persisting through June. The results are consistent with findings in the US, as discussed by Couch et al. (2020).

**Fig. 10:**
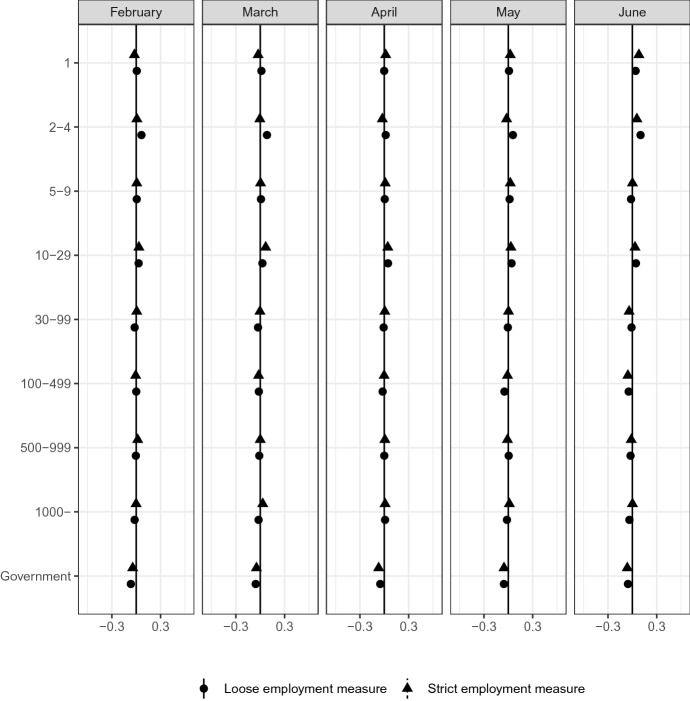
Firm size *Source*: The Labor Force Survey (MIC). *Notes*: This figure shows the results of classification analysis in terms of firm size in the last month. The difference of the bottom 5% and other groups are estimated from Eq. 7 by the difference-in-means estimator using the data from February 2019 to June 2020. Each dot is a point estimate, and the bars are the 95% confidence intervals

Another concern is heterogeneity regarding firm size and type. In general, employment in small- and medium-sized firms is more unstable than that in large firms. Employment by the government may be more stable than that by private firms. Figure 10 checks the distribution of firm size and type in the most affected group and other groups. There are no clear differences between the most affected group and other groups, which implies that our data find no clear heterogeneity regarding firm size and type.

**Fig. 11:**
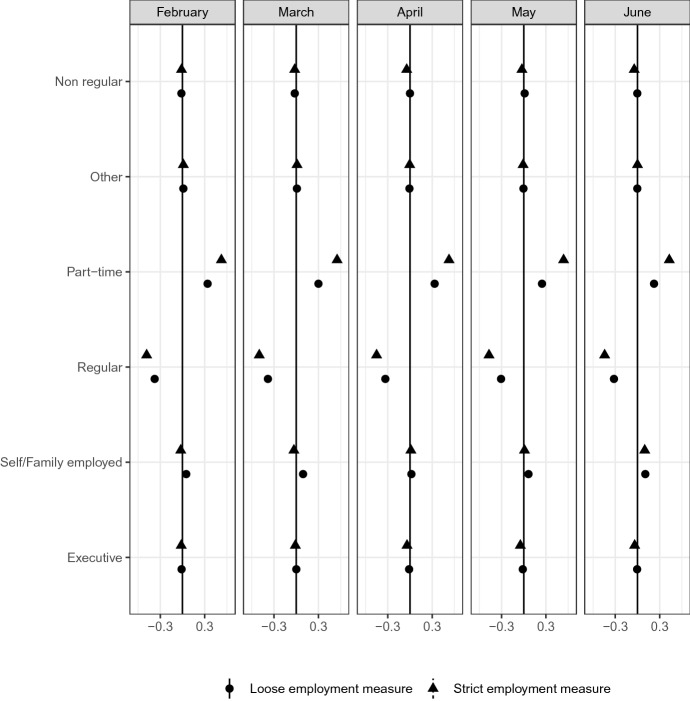
Employment contract type *Source*: The Labor Force Survey (MIC). *Notes*: This figure shows the results of classification analysis in terms of respondent’s employment contract type in the last month. The difference of the bottom 5% and other groups are estimated from Eq. 7 by the difference-in-means estimator using the data from February 2019 to June 2020. Note that non regular includes contract workers (Keiyaku and Shokutaku in Japanese), dispatched workers (Haken in Japanese). Each dot is a point estimate, and the bars are the 95% confidence intervals

Job stability and security may depend on the type of employment contract. Figure 11 reports the significant heterogeneity regarding employment contracts. The distribution of employment contract type is significantly different between the most affected group and other groups. The most affected group consists of more part-time workers. Moreover, the share of regular workers is smaller in this group than in other groups.

**Fig. 12:**
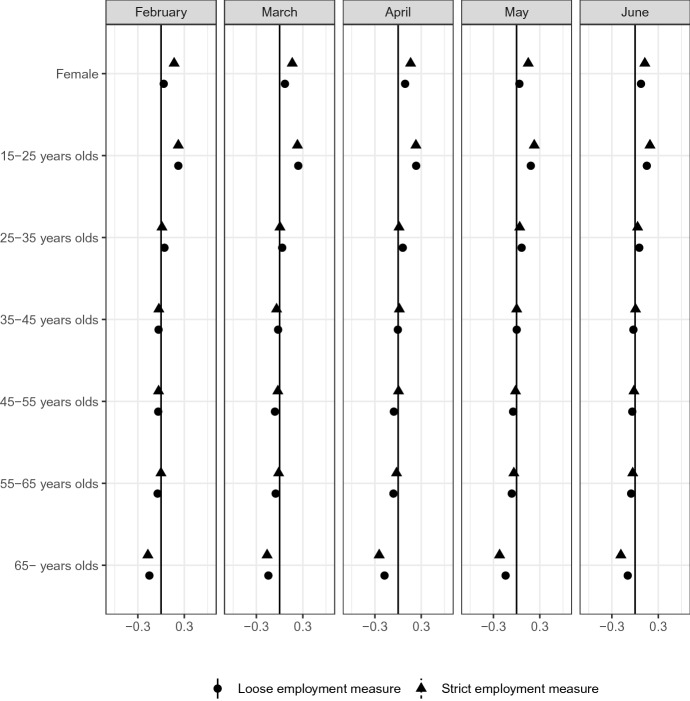
Gender and age *Source*: The Labor Force Survey (MIC). *Notes*: This figure shows the results of classification analysis in terms of gender and age groups. The difference of the bottom 5% and other groups are estimated from Eq. 7 by the difference-in-means estimator using the data from February 2019 to June 2020. Each dot is a point estimate, and the bars are the 95% confidence intervals

The impacts of COVID-19 may vary greatly over some of the demographic background of respondents. (Alon et al. 2020) and (Kikuchi et al. 2021) discuss that female unemployment is greatly increased by COVID-19 than by a regular recession. (Bui et al. 2020) points out the heterogeneity regarding age. Figure 12 also shows the systematic differences in terms of gender and age.

Figure 12 reports the age and gender distributions. The most affected group consists of many young and female respondents. The share of respondents aged 65 years and older is smaller in the most affected group than in other groups. A larger percentage of respondents aged under 25 years are in the most affected group than in other groups.

**Fig. 13:**
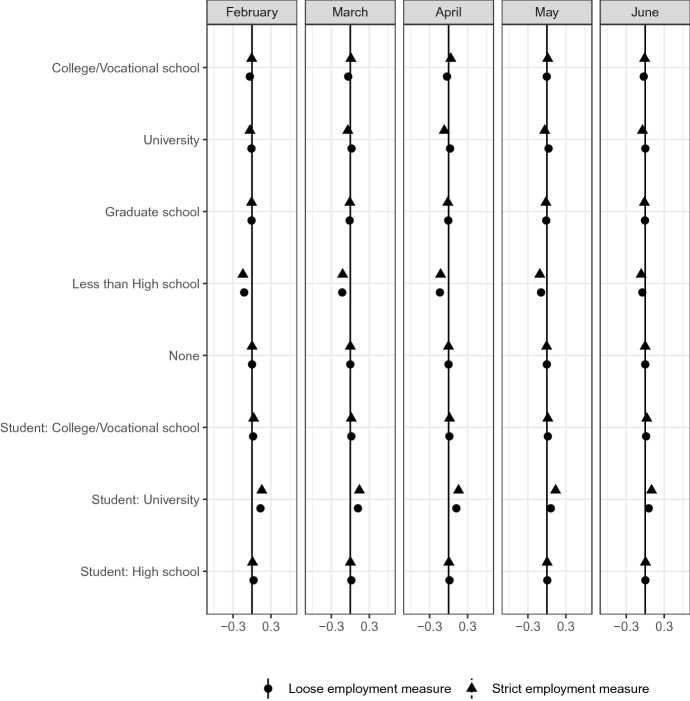
Education status *Source*: The Labor Force Survey (MIC). *Notes*: This figure shows the results of classification analysis in terms of education. The difference of the bottom 5% and other groups are estimated from Eq. 7 by the difference-in-means estimator using the data from February 2019 to June 2020. Each dot is a point estimate, and the bars are the 95% confidence intervals

Figure 13 shows the distribution of education status. First, the share of university students is larger in the most affected group than in other groups. A potential reason for this is that university students working part-time jobs are strongly affected by COVID-19. Second, the share of lower-educated respondents (less than high school) is smaller in the most affected group than in the other groups. Couch et al. (2020) reports the largest unemployment rate of respondents with less than a high school education and high school graduates in April. Our results show that the impacts of COVID-19 are not sorted by educational background in Japan.

**Fig. 14:**
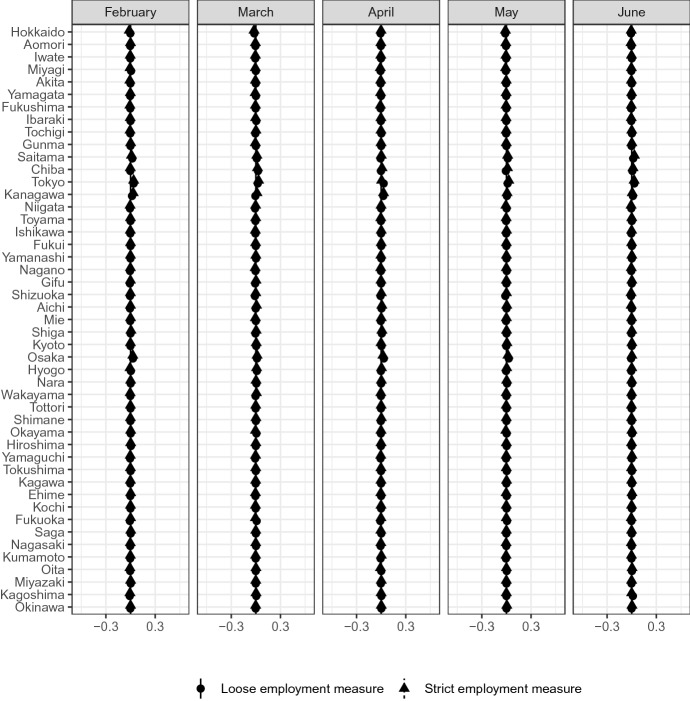
Prefecture *Source*: The Labor Force Survey (MIC). *Notes*: This figure shows the results of classification analysis in terms of prefecture. The difference of the bottom 5% and other groups are estimated from Eq. 7 by the difference-in-means estimator using the data from February 2019 to June 2020. Each dot is a point estimate, and the bars are the 95% confidence intervals

Finally, Fig. 14 reports the geographical distribution. The infection status of COVID-19 geographically varies. The infection rate tends to be higher in urban areas than in rural areas. The impact on the labor market may then also be totally different in different areas. However, Forsythe et al. (2020) shows no clear regional variation in the US; the figure here shows the same holds in Japan. There are no clear differences between the most affected group and other groups. Therefore, there was no clear regional concentration until June 2020, even though the infection status differed across regions.

The above findings characterize the group most affected by the marginal distribution of background characteristics. The difference in conditional distribution may provide additional insights into the heterogeneity of the impacts of COVID-19. The following figures report the difference in family structure among current students and the reason for job search among unemployed workers.

### Family structure among students

**Fig. 15:**
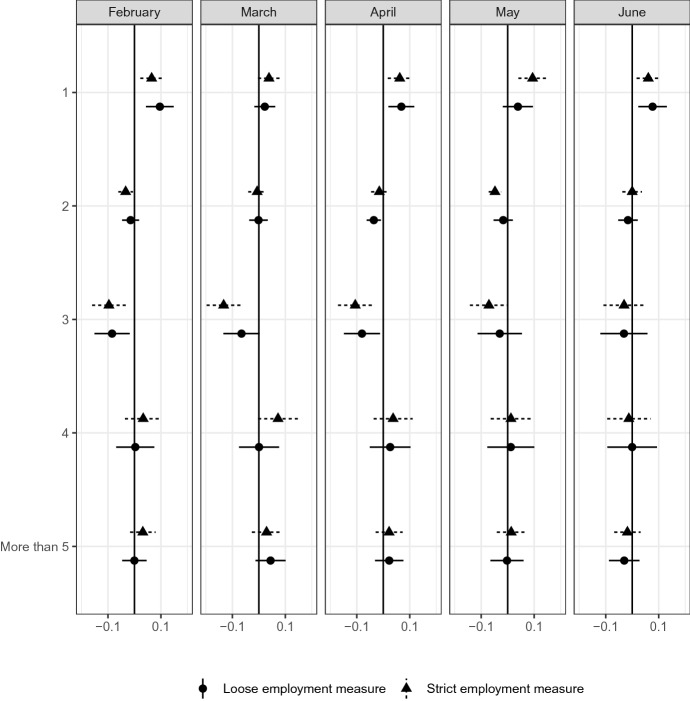
Family *Source*: The Labor Force Survey (MIC). *Notes*: This figure shows the results of classification analysis in terms of family structure. The difference of the bottom 5% and other groups are estimated from Eq. 7 by the difference-in-means estimator using the data from February 2019 to June 2020. Each dot is a point estimate, and the bars are the 95% confidence intervals

Figure 13 reports that current students tend to be strongly affected by COVID-19, the impacts of which may be heterogeneous regarding family structure because their income tends to depend on their parents. Therefore, their family structure affects their income vulnerability to COVID-19 shocks. Figure 15 reports the difference in the number of family members aged 15 years and older among school students.

The figure reports a larger share of single-person households in the most affected group than in other groups. This finding may imply that students who are more vulnerable to economic and social shocks may be more affected by COVID-19 because living alone makes it difficult for families to help one another.

### Reason for job search

Figure 7 shows that the job-finding rate of unemployed workers decreased during COVID-19. The Labor Force Survey includes the reason for job search, which gives us more insight into the heterogeneity among unemployed workers.

**Fig. 16:**
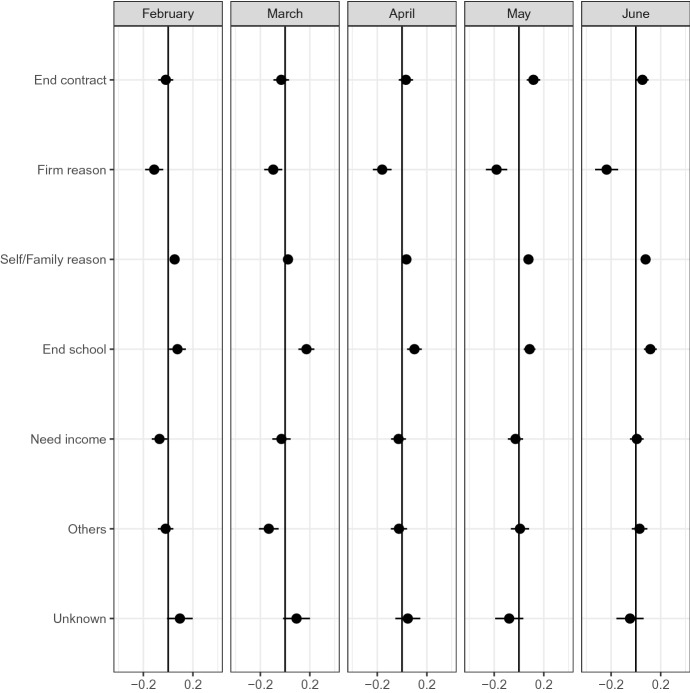
Search reason *Source*: The Labor Force Survey (MIC). *Notes*: This figure shows the results of classification analysis in terms of reason for job search. The difference of the bottom 5% and other groups are estimated from Eq. 7 by the difference-in-means estimator using the data from February 2019 to June 2020. Each dot is a point estimate, and the bars are the 95% confidence intervals

The figure shows a larger share of unemployed workers looking for a job after graduation in the most affected group than in other groups. Moreover, the share of unemployed workers due to firm reasons is smaller in the most affected group than in other groups.

Figures 13 and 16 consistently show the serious impacts of COVID-19 on current students. COVID-19 reduces both the part-time work during school and the job-finding rate after graduation of individuals.

## Conclusion

This paper describes the impacts of the COVID-19 crisis on the Japanese labor market through June 2020. For this purpose, we use a large-scale household survey, the LFS, and the causal machine learning method to detect the heterogeneous impacts of COVID-19.

The first finding is that because of the seasonality in employment status at monthly level, whether we use January as the base month for comparison, as done in most of the studies or whether we use the same month last year as the base comparison group makes a large difference.

Second, we find that whether we include those who are absent from work among the employed or not makes a large difference in the measure of the impact of COVID-19 and its changes.^10^ For example, the most affected group’s employment probability decreased by more than 5 percentage points in April 2020 if workers on leave were included among the employed. If workers on leave were not included among the employed, then the most affected group’s employment probability decreased by more than 20 percentage points. Also, with the strict measure, the employment probability does not seem to be improving toward June, 2020 for many groups.

Third, if we use the strict measure of employment, 25–30% among the employed are adversely affected and that 10% of the employed experienced more than 10% decline in employment probability in April, 2020.

Forth, those who are the most affected by the COVID-19 are those who are unemployed or work part-time in the hotel and restaurant industry and service occupations.

Fifth, younger and female respondents are more affected than are older and male respondents.

Sixth, we observe no clear differences in the impacts of COVID-19 with respect to living location, education status, and firm size among the most affected.

Finally, we discuss the limitations of this paper and directions for future research. First, we studied the impacts of COVID-19 until June 2020. The study period should be extended because important events (e.g., second and third waves of the infection) occurred after June.

Second, an important perspective that we have not explored is the heterogeneity of the impacts of COVID-19 with respect to detailed job characteristics. In particular, many papers (e.g., Dingel and Neiman 2020; Kawaguchi and Motegi 2020; Avdiu and Nayyar 2020) emphasize the heterogeneity of the impacts of COVID-19 by whether the job can be home-based or requires face-to-face interaction.

Third, the LFS provides detailed records of employment history, even though this paper focuses on employment status in the previous month. This detailed employment history may be helpful for understanding the heterogeneity of the impacts of COVID-19.

Finally, in this study, the impacts of COVID-19 are measured using employment status. Whether employed individuals are affected by reduced working hours or reduced wages needs to be examined and is left for future studies.

## Footnotes

1 The only study we know of which uses the same month different year as the base comparison group is Lemieux et al. (2020) that studies the Canadian Labor market.

2 Source: https://corona.go.jp/dashboard/

3 See Takaku and Yokoyama (2021) for the details of these school closures and their impact on child development and other various outcomes.

4 Kubota et al. (2020) summarizes the details of this benefit and analyzes the response of households in terms of their spending.

5 Kawaguchi et al. (2020) focuses on the subsidy policy according to the size of the establishment and evaluates the impact of subsidies on production activities.

6 These series of policies and financial scales for COVID-19 measures in the first half of 2020 are well summarized in Ando et al. (2020)

7 The challenge in this analysis is how to successfully predict employment in 2020: trends may differ by age and gender, such as the tendency for the employment rate of women and the elderly to increase in the 2010s (Kawaguchi and Mori 2019). Table 1 examines the stability of the results with a model that allows for different trends by gender, age, and gender and age. It can be seen that our results are stable for each of the specifications, with April 2020 having the largest impact.

8 In our analysis, it is also crucial to consider which years are used in the prediction. The period around the Global Financial Crisis and the Great East Japan Earthquake are not included in this analysis because the trend of the employment rate differs greatly from that after those events. We have also examined the changes in the results depending on which year is used for forecasting after 2013. Table 2 summarizes the results, showing that the impact on the labor market is more pronounced when data from 2015, which is closer to 2020, is included in the predictions. This is because the rate of improvement in the labor market conditions is somewhat higher more recently between 2013 and 2019, so projections using an older time period will lower the predicted outcome a bit. However the main findings here are not affected very much by which year we use as the initial year for prediction. For example, looking at the Loose employment results for April 2020, the coefficient has changed from −0.0094 to −0.0149, but given that the level of the employment rate is 0.6040, this is not a substantial swing that would change the results.

9 The conditional average difference can be interpreted as the conditional average treatment effect with the conditional independence assumption. Wager and Athey (2018) and Athey et al. (2019) then refer to their proposed algorithm as the causal forest.

10 This point has been observed also in the US. See for example Bartik et al. (2020).
